# Enhanced influenza vaccines impact effectiveness in individuals aged 65 years and older, Denmark, 2024/25 influenza season up to 4 March 2025

**DOI:** 10.2807/1560-7917.ES.2025.30.12.2500174

**Published:** 2025-03-27

**Authors:** Hanne-Dorthe Emborg, Palle Valentiner-Branth, Ramona Trebbien, Amanda Bolt Botnen, Tyra Grove Krause, Bolette Søborg

**Affiliations:** 1 Department of Infectious Disease Epidemiology and Prevention, Statens Serum Institut, Copenhagen, Denmark; 2 Department of Virology and Microbiological Preparedness, Statens Serum Institut, Copenhagen, Denmark; 3 Epidemiological Infectious Disease Preparedness, Statens Serum Institut, Copenhagen, Denmark

**Keywords:** Influenza, vaccine effectiveness, type specific, ≥ 65 years

## Abstract

During the 2024/25 influenza season, enhanced and standard-dose influenza vaccines were available for individuals aged 65 and older. Compared with the standard-dose quadrivalent influenza vaccine (QIV), the adjuvanted QIV was significantly more effective, with an overall vaccine effectiveness (VE) of 48% (95% CI: 42–52) vs 33% (95% CI: 24–41) when considering both non-hospitalised and hospitalised patients. The high-dose QIV demonstrated similar effectiveness to the adjuvanted QIV. These findings support the inclusion of enhanced influenza vaccines in future vaccination programmes.

In Denmark, people aged 65 and older were offered free seasonal influenza vaccination from 1 October to 31 December 2024. For the first time, three different quadrivalent influenza vaccine (QIV) types were available in parallel: a standard-dose QIV, an adjuvanted QIV, and a high-dose QIV. This situation provided a unique opportunity to compare vaccine-type-specific effectiveness (VE) during a high-intensity season dominated by influenza A(H1N1)pdm09 but with substantial co-circulation of A(H3N2).

## Surveillance of seasonal influenza

According to national guidelines, patients belonging to risk groups, including people aged 65 and older, who present with influenza-like illness (ILI) at a general practitioner or with ILI and/or lower respiratory symptoms at hospitals, should be swabbed and tested by reverse transcription (RT)-PCR for influenza A and B viruses (https://www.infmed.dk/guidelines#influenza_(2024).pdf). The test results are registered in real-time in the Danish National Microbiology Database [1].

In week 49 of 2024, Denmark officially declared the start of the 2024/25 influenza season, reporting 161 cases of influenza A and 17 cases of influenza B. In week 9 of 2025, the number of weekly cases had increased to 2,527 for influenza A and 642 for influenza B.

## Influenza vaccines used in the 2024/25 season

All three vaccines offered were quadrivalent [2]. People aged 70 years and older received an adjuvanted QIV (Fluad Tetra, Seqirus Netherlands B.V.), while those aged 65–69 years were offered a standard-dose QIV (Influvac Tetra, Viatris; or Vaxigrip Tetra, Sanofi Winthrop Industrie). In addition, a subset of people aged 65 and older took part in a study where they were randomly allocated either a high-dose QIV (Efluelda Tetra, Sanofi Winthrop Industrie) or a standard-dose QIV. Vaccination status and vaccine type administered to each individual were obtained from the Danish Vaccination Register [3].

**Figure 1:**
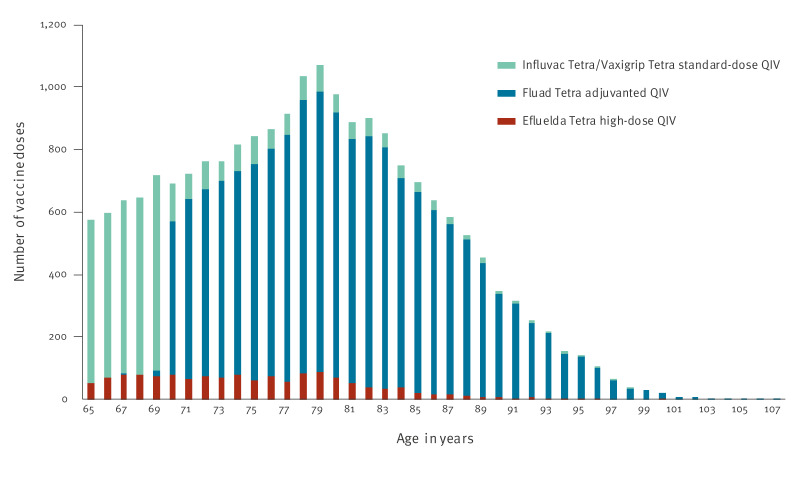
Distribution of vaccine types by age in people aged 65 and older vaccinated against influenza, Denmark, 1 October–31 December 2024 (n = 20,615)

The distribution of the three types of QIV influenza vaccine administered to people aged 65 years and older by age is presented in Figure 1. Among 20,615 vaccinated people aged 65 years and older included in this study, 73.7% (n = 15,197) received the adjuvanted QIV Fluad Tetra, 19.5% (n = 4,010) received a standard-dose QIV of either Influvac Tetra or Vaxigrip Tetra and 6.8% (n = 1,408) received the high-dose QIV Efluelda Tetra.

QIV: quadrivalent influenza vaccine.

Number of people receiving each vaccine: standard-dose QIV of either Influvac Tetra or Vaxigrip Tetra (n = 4,010); adjuvanted QIV Fluad Tetra (n = 15,197); high-dose QIV Efluelda Tetra (n = 1,408).

## Vaccine effectiveness estimation

For vaccine effectiveness (VE) estimation, we defined cases as patients testing positive for either influenza A or B virus by RT-PCR, and controls as those testing negative for both influenza A and B. We obtained information on hospital admissions and underlying chronic conditions from the National Patient Register using the unique person-identifier that all Danish citizens receive at birth or immigration [4]. Patients admitted to the hospital for 12 h or more were categorised as hospitalised while patients with none or less than 12 h hospital contact were categorised as non-hospitalised patients. Patients with one or more underlying chronic diseases, i.e. cardiovascular diseases, diabetes, haematological cancers, immune diseases, kidney diseases, neurological diseases, obesity and respiratory diseases, within the past 5 years were categorised as having chronic disease.

Patients were defined as vaccinated if they received the vaccine at least 14 days before date of sample. Patients with 0–14 days between the vaccination date and sample date were excluded. Interim VE was estimated using the test-negative case–control design as VE = (1 – odds ratio (OR) of vaccination between cases and controls) x 100 using logistic regression, with adjustments for age (5-year groups), sex, testing week and underlying chronic conditions. The combined analyses including hospitalised and non-hospitalised patients were also controlled for hospital admission.

## Characteristics of cases and controls

From 1 October 2024 to 4 March 2025, we identified 3,340 influenza A cases, of these 1,403 (42.0%) were non-hospitalised. Among influenza A cases, chronic conditions were more common in hospitalised patients at 76.1% (n = 1,475) compared with non-hospitalised patients at 54.7% (n = 768). Overall, non-hospitalised cases and controls were more frequently vaccinated at 68.8% and 69.7%, respectively, compared with hospitalised cases and controls at 60.3% and 59.0%, respectively (Table 1). Influenza B virus was only detected in 50 patients aged 65 and older and therefore VE against influenza B was not calculated.

**Table 1:** Influenza A cases and controls in the 2024/25 interim seasonal vaccine effectiveness analysis of non-hospitalised and hospitalised patients aged 65 and older, Denmark, 1 October 2024–4 March 2025 (n = 32,937)

Characteristics	Non-hospitalised patients				Hospitalised patients			
	(n = 11,006)				(n = 21,937)			
	Influenza A cases		Controls		Influenza A cases		Controls	
	n	%	n	%	n	%	n	%
Total	1,403	12.7	9,603	87.3	1,937	8.8	20,000	91.2
Age group (years)								
65–69	421	30.0	2,428	25.3	305	15.7	2,547	12.7
70–74	326	23.2	2,156	22.5	327	16.9	3,158	15.8
75–79	276	19.7	2,117	22.0	411	21.2	4,488	22.4
80–84	215	15.3	1,603	16.7	430	22.2	4,431	22.2
85–89	123	8.8	884	9.2	298	15.4	3,280	16.4
≥ 90	42	3.0	415	4.3	166	8.6	2,096	10.5
Sex								
Female	721	51.4	5,472	57.0	937	48.4	9,675	48.4
Male	682	48.6	4,131	43.0	1,000	51.6	10,325	51.6
Chronic conditions^a^								
Yes	768	54.7	5,629	58.6	1,475	76.1	16,196	81.0
No	635	45.3	3,974	41.4	462	23.9	3,804	19.0
Vaccination status^b^								
No	438	31.2	2,911	30.3	769	39.7	8,210	41.1
Yes	965	68.8	6,692	69.7	1,168	60.3	11,790	59.0

^a^ Cardiovascular diseases, diabetes, haematological cancers, immune diseases, kidney diseases, neurological diseases, obesity and respiratory diseases.

^b^ Individuals were considered vaccinated if they had received one of the 2024/25 influenza vaccines at least 14 days before being tested.

## Virus characteristics

**Figure 2:**
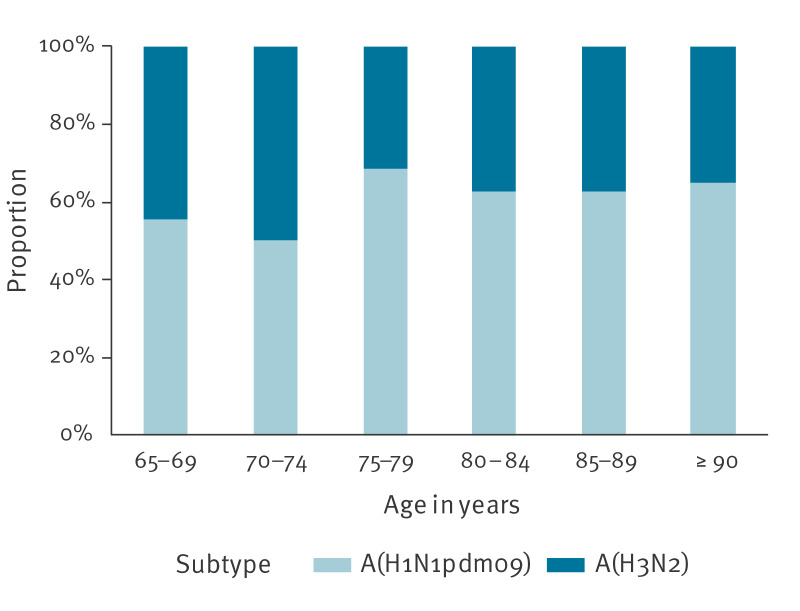
Proportion of influenza A subtypes by 5-year age groups, Denmark, 2024/25 season up to 4 March 2025 (n = 319)

A random subsample of the influenza virus positive samples was subtyped and genetically characterised at the National Influenza Center at Statens Serum Institut (SSI). The influenza A virus subtypes A(H1N1)pdm09 and A(H3N2) have been co-circulating with an increasing proportion of A(H3N2) since January 2025. By 4 March 2025, the overall distribution was 53% A(H1N1)pdm09 and 47% A(H3N2) among 2,073 influenza A virus-positive samples submitted for subtyping at SSI. In the subset of 319 influenza A viruses subtyped and included in this study, 191 (59.9%) were A(H1N1)pdm09 (Figure 2).

## Impact of enhanced influenza vaccines

Compared with the standard-dose QIV (33%; 95% confidence interval (CI): 24–41), the adjuvanted QIV was significantly more effective, with overall VE estimates of 48% (95% CI: 42–52; p < 0.0001) when combining non-hospitalised and hospitalised patients. The high-dose QIV, with an overall VE of 50% (95% CI: 38–59), showed similar effectiveness to the adjuvanted QIV. Stratified analyses of non-hospitalised and hospitalised patients yielded similar VE estimates (Table 2). As the adjuvanted QIV was only offered to people aged 70 years and older we performed and additional VE analysis that was restricted to this age group. This analysis gave comparable results (data not shown).

**Table 2:** Adjusted influenza vaccine effectiveness by vaccine type and patient setting against laboratory-confirmed influenza A in adults 65 years and older, Denmark, 2024/25 season up to 4 March 2025 (n = 32,943)

Patient setting	Vaccine name	Vaccine type	Cases			Controls			VE	95% CI	p value enhanced vs standard^a^
			All	Vacc.	%	All	Vacc.	%			
All	Efluelda Tetra	High-dose QIV	1,332	125	9.4	12,404	1,283	10.3	50	38–59	0.005
	Fluad Tetra	Adjuvanted QIV	2,685	1,478	55.1	24,840	13,719	55.2	48	42–52	< 0.0001
	Influvac Tetra/Vaxigrip Tetra	Standard-dose QIV	1,737	530	30.5	14,601	3,480	23.8	33	24–41	Ref.
Non-hospitalised	Efluelda Tetra	High-dose QIV	516	78	15.1	3,580	669	18.7	50	35–62	0.03
	Fluad Tetra	Adjuvanted QIV	1,004	566	56.4	7,157	4,246	59.3	49	40–57	< 0.0001
	Influvac Tetra/Vaxigrip Tetra	Standard-dose QIV	759	321	42.3	4,688	1,777	37.9	31	17–42	Ref.
Hospitalised	Efluelda Tetra	High-dose QIV	816	47	5.8	8,824	614	7.0	53	35–66	0.05
	Fluad Tetra	Adjuvanted QIV	1,681	912	54.3	17,683	9,473	53.6	47	41–53	0.001
	Influvac Tetra/Vaxigrip Tetra	Standard-dose QIV	978	209	21.3	9,913	1,703	17.2	36	23–47	Ref.

CI: confidence interval; QIV: quadrivalent influenza vaccine; Ref.: reference; Vacc.: vaccinated; VE: vaccine effectiveness.

^a^ The effectiveness of the enhanced vaccines high-dose QIV and adjuvanted QIV are compared with the effectiveness of the standard-dose QIV.

## Discussion

In previous seasons in Denmark, e.g. 2021/22 and 2022/23, VE against influenza A was lower in people aged 65 years and older than in younger groups [5,6]. This was not observed in 2024/25 season, when enhanced vaccines were introduced for older adults [7]. Interim results from this season in Canada, where enhanced vaccines were offered to people aged 65 and older, showed similar or slightly higher VE against influenza A compared with younger age groups [8].

Earlier this season, as part of a European study, we provided Danish interim VE estimates for non-hospitalised and hospitalised patients aged 65 and older for the three vaccines combined. The VE was estimated at 55% (95% CI: 44–64) for non-hospitalised patients and 55% (95% CI: 47–62) for hospitalised patients [7], which was higher than the estimates for any of the three vaccines individually shown in the present study. In the European study, we also found that the VE was significantly higher against A(H1N1) than A(H3N2) in all ages in primary care setting. As a result, the shift in the distribution of these two influenza subtypes observed in Denmark since January will impact the estimated VE. In addition, fewer data were available for the European study, which can also influence the VE estimates.

## Conclusion

The introduction of adjuvanted QIV significantly improved influenza protection in individuals aged 65 and older in Denmark compared with standard-dose QIV. High-dose QIV and adjuvanted QIV showed similar effectiveness. These findings highlight the public health benefits of enhanced vaccines in preventing influenza and reducing severe outcomes in older people, supporting its inclusion in future vaccination programmes.
